# World AIDS Day — December 1, 2019

**DOI:** 10.15585/mmwr.mm6847a1

**Published:** 2019-11-29

World AIDS Day, observed annually on December 1, draws attention to the status of the human immunodeficiency virus/acquired immunodeficiency syndrome (HIV/AIDS) epidemic. Approximately 37.9 million persons worldwide are living with HIV infection, including 1.7 million persons newly infected in 2018 (*1*).

With support from the U.S. President’s Emergency Plan for AIDS Relief (PEPFAR), several African countries are on track to achieve HIV epidemic control. In 2017, an estimated 1,020,419 persons in the United States and dependent areas were living with diagnosed HIV infection; 37,832 new cases were diagnosed in 2018 (*2*). The aim of the U.S. Department of Health and Human Services’ proposed Ending the HIV Epidemic: A Plan for America initiative (*3*) is to end the U.S. HIV epidemic within 10 years.

Through global efforts, including PEPFAR, in 2018, 23.3 million persons worldwide received antiretroviral therapy. A report in this issue of *MMWR* describes the status of implementation of HIV case-based surveillance systems in 39 PEPFAR-supported countries (*4*).
