# *Erratum:* Vol. 68, No. 47

**DOI:** 10.15585/mmwr.mm685152a4

**Published:** 2020-01-03

In the report “World AIDS Day — December 1,” on page 1089, the fourth reference should have read as follows:

4. **Holmes JR**, Dinh T-H, Farach N, et al. Status of HIV case-based surveillance implementation in 39 U.S. PEPFAR-supported countries, May–July 2019. MMWR Morb Mortal Wkly Rep 2019;68:1089–95.

